# Nonverbal Short-Term Serial Order Memory in Autism Spectrum Disorder

**DOI:** 10.1037/abn0000203

**Published:** 2016-10

**Authors:** Dermot M. Bowler, Marie Poirier, Jonathan S. Martin, Sebastian B. Gaigg

**Affiliations:** 1 Devon Autism and ADHD Service, Department of Psychology, City, University of London; 2 Devon Partnership NHS Trust, Devonshire, United Kingdom; 3 Department of Psychology, City, University of London

**Keywords:** autism spectrum disorder, short-term memory, serial order

## Abstract

To clarify the role of item and order memory in the serial recall of adults with autism spectrum disorder (ASD), we carried out 2 experiments in which adults with ASD and comparison participants matched on chronological age and verbal IQ saw sequences of 7 dots appear sequentially in a 3 × 4 grid. In Experiment 1 (serial recall), they had to recall the locations and the presentation order of the dots by tapping locations on an empty grid. In Experiment 2, (order reconstruction) the studied dots were provided at test and participants had to touch them in their order of appearance at study. Experiment 1 revealed diminished item and order recall in the ASD group; Experiment 2 revealed diminished order recall only when verbal IQ was controlled. The results support the view that people with ASD have particular difficulty with serial order recall but may use their language ability to achieve better serial recall performance.

## General Scientific Summary

When asked to recall a sequence of dot locations in order, adults with autism spectrum disorder (ASD) make more errors in recalling the order of the locations than do matched typical individuals. This difficulty is less evident when the dot locations are provided at test, and participants simply have to reconstruct the order in which they originally appeared. Together with parallel findings using verbal material, there appears to be a general (cross-domain) deficit in order processing in individuals with a diagnosis of ASD. There is also some evidence that the ASD participants relied on language capability to accomplish this nonverbal task.

Although not part of the diagnostic criteria for autism spectrum disorder (ASD; American Psychiatric Association, 2000, 2013), the condition shows a characteristic pattern of memory difficulties (see Boucher & Bowler, 2008; Boucher, Mayes, & Bigham, 2012 for reviews). Nondeclarative, or implicit, forms of memory are generally intact, as is long-term, declarative memory when test procedures provide clues to aid retrieval. However, performance tends to be diminished on tasks such as free recall, because there is less support for retrieval at test (Bowler, Gardiner, & Berthollier, 2004; Bowler, Gaigg, & Gardiner, 2008, 2010), a phenomenon labeled by Bowler and colleagues (Bowler et al., 2004; Bowler, 2007) as the task-support hypothesis. Greater difficulty is also reported on more complex memory tasks (Williams, Minshew, & Goldstein, 2015), especially those involving relational processing of information (Gaigg, Gardiner, & Bowler, 2008; Ring, Gaigg, & Bowler, 2016; Maister, Simons, & Plaisted-Grant, 2013) and the processing of context (Greimel et al., 2012; O’Shea, Fein, Cillessen, Klin, & Schultz, 2005). Research into memory over the short term in ASD has tended to employ the working-memory framework (Baddeley & Hitch, 1974), which has both memory and executive-function (EF) components, and the consensus is that any working-memory difficulties found tend to reflect EF rather than memory difficulties (Kercood, Grskovic, Banda, & Begeske, 2014). These findings, coupled with early reports of unimpaired memory span in ASD (O’Connor & Hermelin, 1967; Hermelin & O’Connor, 1967, 1970) have led, until relatively recently, to a widely held view that memory over the short term is intact in this population.

Poirier, Martin, Gaigg, and Bowler (2011) questioned this view, noting that many of the early studies of short-term or immediate memory (STM) were carried out on samples matched on digit span, thereby increasing the likelihood of a nonsignificant group difference. To overcome this design limitation, Poirier et al. (2011) carried out three experiments comparing performance of adults with ASD and IQ scores within the normal range with a control group of typically developed adults. We consider it crucial to note that their ASD and comparison samples, although matched on overall cognitive ability, were not matched on digit span. In their first experiment, participants were asked to recall sequences of visually presented digits either in the order in which they had appeared at study or in reverse order. The results revealed that the ASD group recalled significantly fewer items in order than did the comparison group, but that this was because the ASD group made significantly more order errors in recall; the group difference in number of items correct, irrespective of order, was not significant. The second experiment used the same procedure, but increased demands on item memory by using words instead of digits as to-be-remembered items. The pattern of results was the same as for the first experiment. Poirier et al.’s (2011) third experiment tested the capacity of participants with ASD and comparison participants to recognize changes in the order of studied list items. Participants were presented with sequences of six 2- and 3-syllable words followed by a recognition test on which half the trials swapped the presentation of two of the study-list elements. The results revealed that the ASD group showed significantly reduced detection of changed lists at test. Poirier et al. concluded that verbal STM in ASD is characterized by a specific difficulty with the processing of the order of remembered material, a conclusion that is further supported by the work of Gaigg, Bowler, and Gardiner (2014), who reported intact memory for semantic order of elements (the actual chronological sequence of historical figures) with diminished memory for their episodic order (an arbitrary, experimenter-determined order).

Poirier et al.’s (2011) finding of intact item memory but diminished order memory may be a consequence of the verbal nature of the stimuli or of the difficulties that many individuals on the autism spectrum have with temporal processing (see Boucher, 2001, 2012). Studies of long-term memory for verbal material in ASD bear out these conjectures by showing diminished relational and intact item-specific processing (Gaigg et al., 2008; Maister et al., 2013), as well as diminished temporal reproduction (Falter, Noreika, Wearden, & Bailey, 2012; Maister & Plaisted-Grant, 2011; Martin, Poirier, & Bowler, 2010). These considerations raise the question of whether the pattern reported by Poirier et al. (2011) is limited to verbal material or is a more universal characteristic of autistic STM. Several studies have shown that children and adults with ASD have lower spatial STM or working-memory span than appropriate comparison groups (Jiang et al., 2014; Joseph et al., 2005; Williams et al., 2005; Zinke et al., 2010, see Kercood et al., 2014 for review). Diminished serial recall in a group of nonintellectually disabled children with ASD was found by Williams et al., (2005), who used the Finger-Windows test from the *Wide Range Assessment of Memory and Learning* (WRAML, Sheslow & Adams, 1990), in which participants had to recall through which holes in a card the examiner pushed a pencil. However neither this, nor any of the other studies reviewed by Kercood et al. (2014), attempted to measure item and order memory separately.

The two experiments described here were designed to address these issues. We used a location-memory test modeled on the dots test (Jones, Farrand, Stuart, & Morris, 1995; Parmentier & Andrés, 2006; Parmentier, Elford, & Maybery, 2005). Participants were shown a grid in which a sequence of dots appeared on a touch-sensitive screen, with each dot disappearing before the next one appeared. In Experiment 1, participants had to tap the grid in the locations and in the order in which the dots had appeared at study. In the test phase of Experiment 2, the dots were presented together on the grid, with participants having to touch them in the order in which they had appeared at study. Assuming participants with ASD have an order-memory difficulty that crosses domains, our prediction was that participants with ASD would make more order errors than comparison participants in Experiment 1, which required both item and order recall. Based on the hypothesis that there is a general order-recall difficulty in ASD and considering data with verbal materials (i.e., Poirier et al.’s [2011] Experiment 3), a significant difference between groups was also expected in Experiment 2, in which location memory was supported by providing the dot locations at test, but the temporal order of the locations still had to be remembered.

## Experiment 1

### Method

#### Participants

Twenty individuals with ASD (14 men) and 20 typical individuals (15 men) took part. Participants were group-matched on the Verbal IQ scale (VIQ; with the digit span subtest score removed) of the *Wechsler Adult Intelligence Scale–R/III* (Wechsler, 1997) and groups did not differ on the Performance IQ scale (PIQ), full-scale IQ, or age (see Table 1). All ASD participants had received their diagnoses by qualified clinicians through the UK National Health Service and were included in the study only if the written diagnosis contained sufficient information to justify meeting criteria. Individuals trained to research reliability standards on the Autism Diagnostic Observation Schedule (ADOS, Lord et al., 1989, see Table 1 for scores) administered this instrument to all but two ASD participants. For all but three participants, the Communication and Reciprocal Social domain scores were above the recommended threshold for an autism spectrum diagnosis. Although the remaining participants scored below one or both of these thresholds, they were retained in all analyses, as their clinical records clearly confirmed their diagnosis. Moreover, administration of the Autism Spectrum Questionnaire (Baron-Cohen et al., 2001) provided further corroboration of the ASD participants’ difficulties in reciprocal social communication and also helped rule out such difficulties in the comparison participants. The comparison group was recruited via local newspaper advertisements, and brief interviews ensured that no participant had a history of neuropathology or psychiatric illness. Individuals gave their informed consent to take part in the study and were paid standard university fees for their participation.[Table-anchor tbl1]

#### Materials

A custom program was designed using Microsoft Visual Basic 6 to run the experiment on a Hewlett–Packard laptop computer with a touch-sensitive screen. Participants responded throughout the experiment by tapping on the screen with a touch-screen stylus.

The stimuli consisted of 35 fixed sequences of seven dots (or paths) that were presented in random order for each participant. This sequence length is typical of this task and usually does not involve any floor or ceiling effects (Jones et al., 1995; Parmentier et al., 2005). Moreover, the sequence length is comparable to those used in verbal tasks, making this paradigm a better visuospatial analog of immediate verbal recall than other tasks. Each of the paths was the same length, that is, if each sequence of dots was conceived as a continuous line and measured, they would all be of identical length. Each path also contained one cross, that is, where the hypothetical “line” went across itself; this contributed to equating difficulty across trials (Parmentier & Andrés, 2006; Parmentier et al., 2005). The path length was chosen by generating a sample of all possible paths (regardless of length) and then inspecting the distribution of lengths. The length selected was close to the mean path length, and contained a large number of possible paths from which to draw a random sample. From this sample, paths that contained lines that overlapped (i.e., where the line segments were superimposed) were excluded, as were all paths with either no crossings or more than one crossing. From this sample, 35 paths were randomly selected and visually checked to make sure there were no irregularities.

#### Procedure

On-screen instructions outlined the experiment, and participants were asked if they had any questions. They were then shown two trials in which the experimenter demonstrated the task. Following these two trials, participants were allowed to practice the task for two trials, and were asked again if they understood the task. On each trial, a sequence of seven dots was presented within a 3 × 4 grid that remained visible on the screen during presentation and recall. Dots never appeared twice in the same location within a given trial sequence. Each dot was presented for 600 ms, after which it disappeared and there was a gap of 200 ms before the presentation of the next dot. After all seven dots were presented, the participant was instructed to tap on the grid locations where the dots had appeared in the order in which they had appeared (starting with the first dot in the sequence). When a location was tapped, a dot appeared for 600ms (replicating the context of the presentation stage) although participants were able to select their next location before the dot disappeared. If participants were unsure about the location of a particular dot within the sequence, they were encouraged not to guess, but to tap on a “skip” button at the bottom of the screen, which enabled them to move onto the next dot position. Following the completion of each trial, participants had to tap on a button on the screen to start the next trial.

### Results and Discussion

**Figure 1 fig1:**
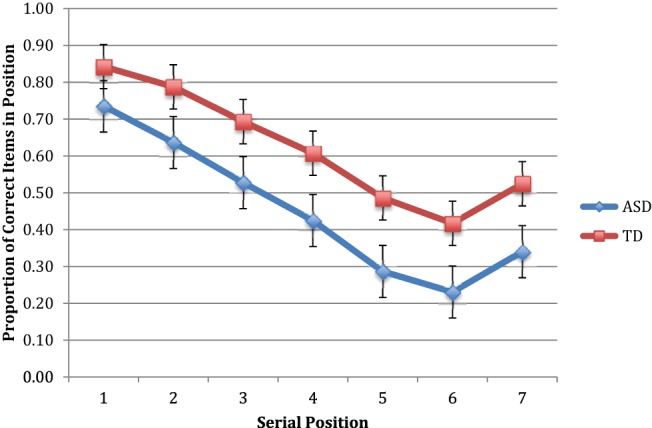
Mean proportions of correct items in position for order recall in Experiment 1 for ASD and Comparison participants. Error bars represent ±1 SEM. See the online article for the color version of this figure.

Results were first scored in terms of number of correct locations reported in order. In other words, to be considered correct, a response had to involve a studied location. In addition, its position in the response sequence had to be the same as in the studied series. Skip responses were counted as omissions and scored as incorrect. Analysis of the average number of correct locations in order (illustrated in Figure 1) using a 2 (group) × 7 (position) repeated-measures ANOVA revealed a significant main effect of group, (*F* = 9.73, *df* = 1,38, *p* = .004, η_p_^2^ = 0.20, 95% CI [0.04, 0.37]). The average proportion of dots recalled in their correct serial position was 0.45 (*SD* = 0.17) for the ASD group and 0.62 (*SD* = 0.18) for the comparison group. Cohen’s *d* statistic was 0.89, 95% CI [0.32, 1.64], indicating a large effect size. There was a main effect of serial position (*F* = 76.01, Greenhouse–Geisser corrected *df* = 1.99, 75.47, *p* = .001, η_p_^2^ = 0.67, 95% CI [0.59, 0.71]), but no Serial Position × Group interaction (*F* < 1, *ns*, η_p_^2^ = 0.16). Prorated VIQ (with digit span removed) correlated with average proportion correct for the ASD (*r* = .57, *df* = 23, *p* < .005), but not for the comparison group (*r* = .37, *df* = 24, *ns*) and PIQ correlated with average proportion correct for both groups (comparison: *r* = .64, *df* = 22, *p* < .001; ASD: *r* = .54, *df* = 24, *p* < .007). Entering VIQ as a covariate left the main effect for group intact (*F* = 11.49, *df* = 1,37, *p* < .003, η_p_^2^ = 0.24, 95% CI [0.06, 0.40]). The Group × Serial Position interaction remained nonsignificant (*F* < 1, *ns*) but the serial position main effect fell just short of significance (*F* = 2.92, Greenhouse–Geisser corrected *df* = 1.98, 73.26, *p* < .06, η_p_^2^ = 0.07, 95% CI [0.01, 0.11]). Entering PIQ as a covariate left the group effect significant (*F* = 10.24, *df* = 1,36, *p* < .004, η_p_^2^ = 0.22, 95% CI [0.05, 0.39]), as did the serial position effect (*F* = 6.58, Greenhouse–Geisser corrected *df* = 2.03, 72.95, *p* < .002, η_p_^2^ = 0.15, 95% CI [0.07, 0.21); the Group × Serial Position interaction remained nonsignificant (*F* < 1, *ns*).[Fig-anchor fig1]

**Figure 2 fig2:**
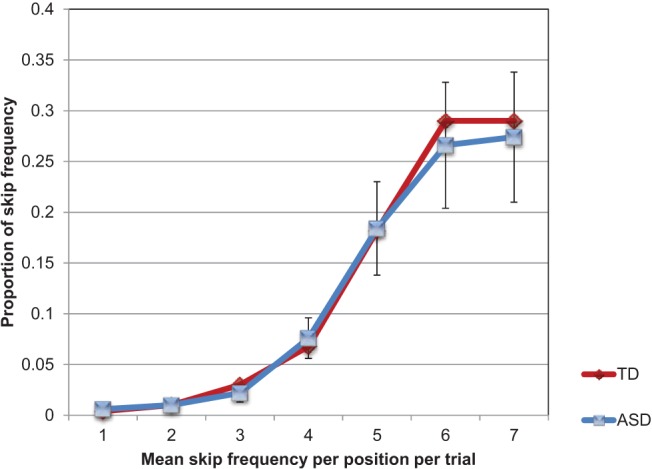
Mean proportions skip frequency per serial position in Experiment 1 for ASD and comparison participants. See the online article for the color version of this figure.

To check whether the group differences just reported could be explained by skip responses, we examined skip frequency per serial position for each group. Assuming skips were omissions (based on typical findings in serial recall tasks; e.g., Henson, 1998), we would expect the frequency of omissions to increase with serial position. Figure 2 illustrates the findings. Very little appears to differentiate the groups. This was confirmed through a 2 (group) × 7 (serial position) ANOVA producing a significant effect of serial position (*F* = 17.96, *df* = 6, 228, *p* < .001, η_p_^2^ = .32, 95% CI [0.21, 0.39]), but no group difference (*F* < 1, *ns*) or Group × Serial Position interaction (*F* < 1, *ns*).[Fig-anchor fig2]

Item errors might also explain the overall group difference in correct recall. Item errors refer to the recall of an incorrect location, that is, a location that was not part of the studied sequence. To examine this possibility, we computed the number of correct locations recalled, without respect to order. In this case, any location recalled that was part of the original list was scored as correct. This type of scoring ignores any transposition in item order (i.e., AB reported as BA) or other types of order error. Average proportion of correct dot locations recalled per trial was significantly lower in the ASD group (*M* = 0.74, *SD* = 0.11) than in the comparison group (*M* = 0.83, *SD* = 0.12), *t* = −2.45, *df* = 38, *p* = .02, Cohen’s *d* = 0.77, 95% CI [0.13, 1.41], suggesting that the ASD group misremembered more of the locations. This difference survived the partialing out of prorated VIQ (*F* = 6.57, *df* = 1,37, *p* < .02, η_p_^2^ = 0.15, 95% CI [0.01, 0.35]) and PIQ (*F* = 6.61, *df* = 1,37, *p* < .02, η_p_^2^ = 0.15, 95% CI [0.01, 0.35]).

Group differences in order recall were also considered (see Saint-Aubin & Poirier, 1999). The difference between the location score (i.e., the overall number of correct items recalled, regardless of order) and the strict serial recall score, provides a straightforward estimate of overall order errors, as the only difference between the two scores is that order errors are considered in strict scoring. The groups also differed on this measure (*t* = 2.14, *df* = 38, *p* = .04, Cohen’s *d* = 0.68, 95% CI [0.15, 1.44], ASD: *M* = 0.29, *SD* = 0.14; comparison: *M* = 0.21, *SD* = 0.11). This group difference in order errors survived the partialing out of prorated VIQ (*F* = 5.03, *df* = 1,37, *p* < .03, η_p_^2^ = 0.12, 95% CI [0.00, 0.32]) and PIQ (*F* = 4.29, *df* = 1,37, *p* < .05, η_p_^2^ = 0.10, 95% CI [0.00, 0.30]).^1^

Analysis of average reaction time (RT) by a 2 (group) × 7 (serial position) ANOVA revealed a significant effect for serial position (*F* = 290.95, Greenhouse–Geisser corrected *df* = 1.16, 41.60, *p* < .001, η_p_^2^ = 0.89, 95% CI [0.87, 0.90]). There was no group difference (*F* = 2.20, *df* = 1,36, *p* = .16, η_p_^2^ = 0.06, 95% CI [0.00, 0.21]) or Group × Serial Position interaction (*F* = 2.23, Greenhouse–Geisser corrected *df* = 1.16, 41.60, *p* < .15, η_p_^2^ = 0.06, 95% CI [0.00, 0.09]).

The finding of diminished order recall replicates the majority of findings on spatial span in individuals with ASD (Kercood et al., 2014), replicates Poirier et al.’s (2011) findings for verbal material, and further supports the idea that STM difficulties in ASD stem, at least in part, from difficulties with time-related contextual processing (Molesworth, Chevallier, Happé, & Hampton, 2015). However, the diminished item recall contrasts with Poirier et al.’s findings for verbal material in similar STM paradigms, perhaps because retrieval and reconstruction processes in verbal STM can rely on prior linguistic knowledge and benefit from the relative distinctiveness of the phonological, lexical, and semantic features of the words. In contrast, the visuospatial task used here involved items that were all identical except for position—and hence had few distinctive features and offered less prior knowledge to call on than words do. This contrast between the relative difficulty that individuals with ASD experience in the recall of specific locations as items and their lack of difficulty with recall of words may reflect their reliance on familiarity to scaffold their word recall over the short term, an account in accord with studies that have shown increased familiarity and diminished recollection, as well as atypical neural signatures for recollection in ASD (Bowler, Gardiner, & Grice, 2000; Bowler, Gardiner, & Gaigg, 2007; Gaigg, Bowler, Ecker, et al., 2015; Massand & Bowler, 2015). Poor performance on visuospatial tasks such as the present one or those reviewed by Kercood et al. (2014), appears to contradict the general view that visuospatial ability constitutes a cognitive strength in people on the autism spectrum (Mitchell & Ropar, 2004). The present task, however, was one of visuospatial *recall*, which taps a memory process well-known to pose difficulties for people with ASD (Boucher et al., 2012). We tested the question of whether order-memory difficulty remains when retrieval of the items is not required by the task in Experiment 2.

## Experiment 2

### Method

#### Participants

Twenty-three individuals with ASD (16 men) and 24 typical individuals (16 men) selected and group-matched as in Experiment 1 took part in this experiment. ADOS (Lord et al., 1989) data were available for 21 of the 23 ASD participants and autism-spectrum quotient data were available for all participants in both groups. Twelve participants with ASD and 13 comparison participants took part in both experiments. Descriptive statistics for both groups are given in Table 1.

#### Materials

The task consisted of 35 fixed sequences of seven dots constructed the same way as for Experiment 1.

#### Procedure

The warm-up and study procedures were identical to those of Experiment 1. At test, when the last dot of each trial had disappeared, all seven dots appeared in the locations in which they had been presented at study and participants were asked to touch each dot in the order in which they had seen it appear at study.

### Results and Discussion

**Figure 3 fig3:**
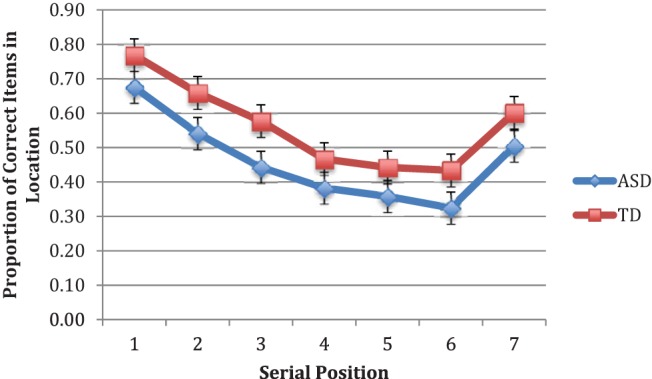
Mean proportions of correct items in position for order reconstruction in Experiment 2 for ASD and Comparison participants. Error bars represent ±1 SEM. See the online article for the color version of this figure.

A first analysis of the average number of locations identified in their correct serial position (set out in Figure 3) using a 2 (group) × 7 (position) repeated-measures ANOVA revealed a significant effect for position (*F* = 58.19, Greenhouse–Geisser corrected *df* = 3.43, 154.54, *p* < .001, η_p_^2^ = 0.56, 95% CI [0.49, 0.61]), but no significant effect either for group (*F* = 2.89, *df* = 1,45, *ns*, η_p_^2^ = 0.06, 95% CI [0.00, 0.22]) or the Group × Position interaction (*F* < 1, *ns*). However, for the ASD group, significant correlations were found between number of correct locations in order and VIQ with digit span removed, *r* = .57, *df* = 23, *p* < .005 and PIQ, *r* = .64, *df* = 22, *p* < .001. For the comparison group, the corresponding correlations were *r* = .37, *df* = 24, *p* < .08 for VIQ and *r* = .54, *df* = 24, *p* < .01 for PIQ. When these variables were entered jointly and separately as covariates into the above ANOVA, only prorated VIQ influenced the outcome, resulting in the group difference becoming significant (*F* = 5.37, *df* = 1,44, *p* < .03, η_p_^2^ = 0.11, 95% CI [0.01, 0.26], adjusted ASD *M* = .46, *SE* = .04; adjusted comparison *M* = 0.55, *SE* = .03). Cohen’s *d* for this difference was moderate at 0.53.[Fig-anchor fig3]

Analysis of average RT by means of a 2 (group) × 7 (serial position) ANOVA revealed a significant effect for serial position (*F* = 46.3, Greenhouse–Geisser corrected *df* = 3.6, 156.62, *p* < .001, η_p_^2^ = 0.51, 95% CI [0.43, 0.55]) with no group difference (*F* < 1, *ns*) or Group × Serial Position interaction (*F* < 1.8, *ns*).

Taken together, these findings suggest that diminished performance in ASD is less in evidence when the task is less reliant on recall; this conclusion needs to be considered with some caution however, as average performance for the ASD group (*M* = 0.45) was identical in Experiments 1 and 2. Comparison group performance dropped by 7% in Experiment 2, relative to Experiment 1 (from 0.62 to 0.55). These variations are likely to be random sampling variations rather than substantive differences that have theoretical meaning. The general picture in both studies showed clear evidence that temporal order processing is more challenging for participants with ASD. Moreover, the observation that order memory correlated more strongly with VIQ for ASD than typically developed (TD) participants further supports the view that ASD participants rely on language ability, and in this case, perhaps through verbally labeling the dot locations and rehearsing these labels to scaffold their performance on serial recall tasks. Once this is factored out by controlling for VIQ, the order-memory difficulty identified in Experiment 1 reappears, suggesting that memory over the short term for the order of events is a pervasive difficulty in ASD evident across domains.

## General Discussion

By showing diminished serial order reproduction in adults with ASD, the present findings demonstrate that memory difficulties for the temporal order of items is a general feature of autistic memory and not limited to the recall of verbal material, as implied by earlier studies such as Poirier et al. (2011). By showing that people with ASD experience difficulty with time-related contextual processing, our findings confirm and extend studies of context memory (Greimel et al., 2012; O’Shea et al., 2005) and temporal cognition (Boucher, 2001; Falter et al., 2012; Maister et al., 2013; Martin et al., 2010).

The diminished item recall in Experiment 1, although in contrast to Poirier et al.’s (2011) findings for verbal material, is in line with the majority of studies of spatial memory in nonintellectually disabled children and adults on the autism spectrum (see Kercood et al., 2014 for review). Our findings extend this literature because the majority of the studies reviewed in Kercood et al. used either variants of the Corsi blocks task (Milner, 1971), the spatial working-memory test from the Cambridge Automated Neuropsychological Test Battery (CANTAB; Robbins et al., 1998), or the Finger-Windows test (Sheslow & Adams, 1990), none of which explicitly dissociates item from order effects. Moreover, those tasks do not compare order recall with order reconstruction in the way that was done here. By dissociating memory for items from memory for order, the findings of the present two experiments represent an important addition to our understanding of nonverbal STM in ASD.

In both experiments, prorated VIQ (i.e., with digit span removed; Wechsler, 1997) correlated with order recall and reproduction for the ASD group only, and diminished order reconstruction was evident in Experiment 2 only when prorated VIQ was controlled statistically. This suggests that ASD participants used their verbal abilities to help them solve this visuospatial task in a way that the comparison participants did not. Atypical recruitment of language-related brain areas in ASD has been reported using verbal (Koshino et al., 2005) and nonverbal (Urbain, Pang, & Taylor, 2015) working-memory (*n*-back) tasks. Taken together with the present findings, these observations point to a potentially important link between language ability, working memory, and memory for serial order in ASD, a link that has also been the object of multiple studies and lively debate in the literature on serial order memory in typical individuals (Bogaerts, Szmalec, Hachmann, Page, & Duyck, 2015; Gathercole, Hitch, Service, & Martin, 1997; Majerus & Boukebza, 2013). Although the present findings cannot tell us whether higher verbal ability is a cause or a consequence of order reconstruction ability in ASD, some speculations can nevertheless be made. Individuals with ASD are known to successfully recruit inner speech to help retain visually presented verbalizable material in STM (Williams, Happé, & Jarrold, 2008), but do not use inner dialogue to assist with complex planning tasks such as the Tower of London (Williams, Bowler, & Jarrold, 2012). The correlation reported here between order reconstruction and verbal ability in the ASD participants suggests that the use of inner speech by ASD participants to support memory in simple STM tasks might extend to order reproduction. Further studies are needed to ascertain the conditions under which individuals with ASD decide to recruit inner speech and whether such conditions could be modified. The outcome of studies such as these could have considerable implications for intervention.

The fact that the present experiments did not provide an unambiguous characterization of the strategies that participants use when performing such experimental tasks, nor did it provide a complete exploration of factors that might influence task performance in both groups, constitutes a limitation of the present study. A further limitation relates to the relatively small sample size, which may have resulted in failure to detect other significant effects or to reveal different patterns of performance resulting from heterogeneity in the ASD sample. Nevertheless, the findings convincingly show that difficulties with memory for serial order are a pervasive feature of autistic memory, whether for verbal or nonverbal material, and that this difficulty is associated with language ability in ways that further experimentation has yet to elucidate.

## Figures and Tables

**Table 1 tbl1:** Age and Psychometric Measures for the Two Participant Groups in Each Experiment

	Experiment 1		Experiment 2	
Variable	ASD group (*n* = 20)	Comparison group (*n* = 20)	ASD group (*n* = 23)	Comparison group (*n* = 24)
Male/Female	14/6	15/5	16/7	16/8
Chronological age (years)				
*M*	35.1	36.5	36.6	36.8
*SD*	13.05	11.16	13.61	11.63
Range	19–56	21–53	19–56	21–54
Full-Scale IQ				
*M*	106.00	106.16	109.48	107.00
*SD*	16.21	19.38	16.46	15.32
Range	81–146	77–139	81–138	77–139
Verbal IQ^a^				
*M*	107.64	106.95	109.87	106.21
*SD*	15.62	15.78	15.07	15.02
Range	79–142	80–137	79–137	80–137
Performance IQ				
*M*	105.46	105.37	107.71	106.58
*SD*	18.99	18.03	16.75	16.37
Range	78–140	72–138	78–135	75–138
Autism-spectrum quotient (AQ)^b^				
*M*	34.55	16	35.43	14.65
*SD*	7.42	6.39	7.84	7.38
Range	22–47	5–28	22–47	4–28
Autism Diagnostic Observation Schedule (ADOS)				
*M*	8.5^c^	—	8.95	—
*SD*	3.54	—	3.80	—
Range	2–16	—	2–17	—
*Note*. ASD = autism spectrum disorder. All other between-group differences were *ns* (max *t* = .83, min *p* = .41).				
^a^ Prorated Verbal IQ excluding the digit span subtest. ^b^ Experiment 1, *t*(38) = 8.47, *p* < .001. Experiment 2, *t*(44) = 9.26, *p* < .001. ^c^ *N* = 18.				

## Footnotes

1 Another measure of order recall frequently used is the proportion of order errors. For this measure, the number of order errors is divided by the number of items correctly recalled, regardless of position. Measuring the proportion of order errors is favored in cases in which the manipulated factor leads to an increase in the number of recalled items, as well as an increase in order errors. As more recalled items can mean more chances to produce order errors, a proportional measure is sometimes preferred. However, in our experiments, relative to the TD group, the ASD group produced *fewer* correct items and a *higher* frequency of order errors. Hence, using a proportional measure would not have been appropriate.
